# A novel pathogenic variant in OSBPL2 linked to hereditary late-onset deafness in a Mongolian family

**DOI:** 10.1186/s12881-019-0781-3

**Published:** 2019-03-20

**Authors:** Ningjin Wu, Husile Husile, Liqing Yang, Yaning Cao, Xing Li, Wenyan Huo, Haihua Bai, Yangjian Liu, Qizhu Wu

**Affiliations:** 1 Affiliated Hospital of Inner Mongolia University for the Nationalities, Tongliao, 028000 China; 2 Xiangya School of Medicine, Central South University, Changsha, 410013 China; 3 Inner Mongolia Engineering Research Center of Personalized Medicine, Tongliao, 028000 China; 4 School of Life Science, Inner Mongolia University, Hohhot, 010021 China; 5 School of Life Science, Inner Mongolia University for the Nationalities, Tongliao, 028000 China; 6 Department of Developmental Biology, Washington University School of Medicine, St. Louis, MO 63110 USA

**Keywords:** Hereditary late-onset deafness, Whole genome sequencing, *OSBPL2* gene, Co-segregation

## Abstract

**Background:**

To investigate the clinical features and the underlying causal gene of a family with hereditary late-onset deafness in Inner Mongolia of China, and to provide evidence for the early genetic screening and diagnosis of this disease.

**Methods:**

Family data were collected to draw a pedigree. Audiological testing and physical examination of the family members were conducted following questionnaire. Genomic DNA was extracted from peripheral blood of 5 family members (3 patients and 2 normal control) and subjected to whole genome sequencing for identifying deafness casual genes. The pathogenic variant in the deafness gene was further confirmed by Sanger sequencing.

**Results:**

The family is composed of a total of 6 generations, with 53 traceable individuals. In this family,19 of them were diagnosed with post lingual deafness with the age of onset between 10 and 40 years, displaying delayed and progressive hearing loss. Patients with hearing loss showed bilateral symmetry and mild to severe sensorineural deafness. The pattern of deafness inheritance in this family is autosomal dominant. Whole genome sequencing identified a novel pathogenic frameshift mutation, c.158_159delAA (p.Gln53Arg fs*100) in the gene *OSBPL2* (Oxysterol-binding protein-related protein 2, NM_144498.2), which is absent from genomic data of 201 unrelated normal subjects. This pathogenic variant was further validated by Sanger sequencing, and was found to co-segregate in this family.

**Conclusions:**

Whole genome sequencing identified a two-nucleotide deletion in *OSBPL*2 (c.158_159delAA) as the pathogenic variant for deafness in the family. Our finding expands the mutational spectrum of *OSBPL*2 and contributes to the pathogenic variant list in genetic counseling for deafness screening.

**Electronic supplementary material:**

The online version of this article (10.1186/s12881-019-0781-3) contains supplementary material, which is available to authorized users.

## Background

Deafness is a defect of the human auditory system, and it is also one of the common diseases that seriously affects human health. Without intervening, the hearing loss in children could lead to delayed speech, difficulty in reading, and lack of confidence, severely affecting their growth and social skills. The deafness could be a result of genetic, environmental and other unknown factors, with genetic factors contributing to the majority cases of deafness. Studies have shown that about 50 to 60% of hearing loss is inherited [1]. The inheritance pattern of deafness includes autosomal dominant inheritance, autosomal recessive inheritance and X-linked [2]. Based on whether there are other systemic or organ disorders, clinical deafness can be divided into non-syndromic deafness and syndrome deafness [3]. Non-syndromic hearing loss (NSHL) constitutes about 70% of congenital hereditary deafness while syndromic hearing loss (SHL) accounts for the other 30%. In developed countries, nearly 80% of the non-syndromic deafness is attributed to genetic factors, of which 75–80% is autosomal recessive, 10–15% is autosomal dominant, and the rest is X-linked or mitochondrial [4]. Among these, autosomal dominant hereditary deafness (also known as autosomal dominant non-syndromic sensorineural hearing loss, DFNA) mostly displays postlingually, late-onset and progressive sensorineural hearing loss [5].

Next-generation sequencing has been demonstrated to be a powerful technology on identifying mutations of Mendelian diseases [6]. In recent years, the application of next-generation sequencing technology has greatly accelerated the identification of new rare disease genes [7].

In this study, we characterized the clinical phenotype of a Mongolian family with hereditary late-onset deafness. To identify the causal gene for deafness in this family, we applied whole genome sequencing analysis to pinpoint *OSBPL*2 (Oxysterol-binding protein-related protein 2) as the disease gene. This is the third family reported with hearing loss due to *OSBPL2* frameshift variants.

## Methods

### Family data and sample collection

**Fig. 1:**
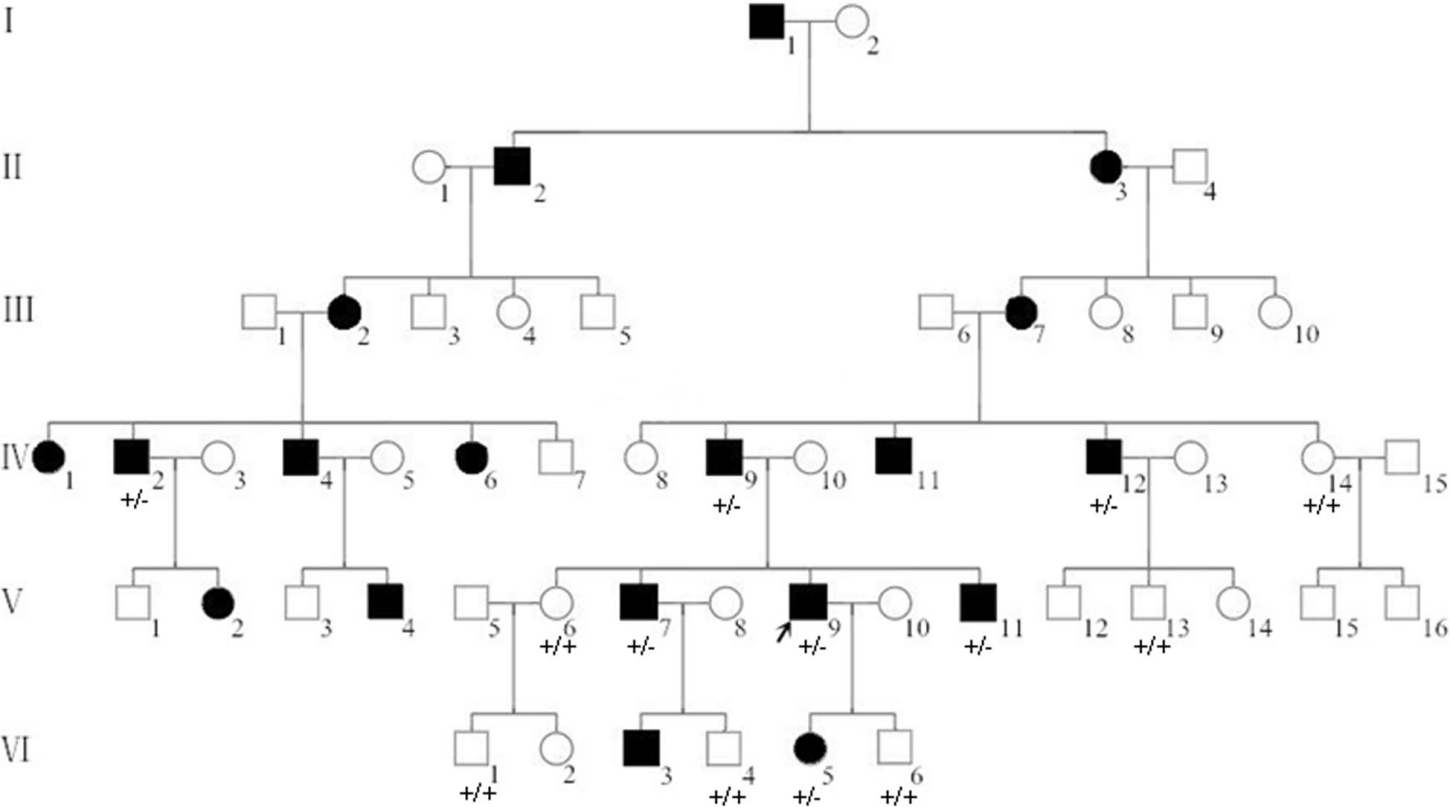
Pedigree and genotypes of the late-onset deafness family. V-9 is the proband. +/−: heterozygous for *OSBPL2*, +/+: wild type for *OSBPL2*. Members with genotypes noted were recruited for this study

The affected family with 19 cases of deafness (12 males and 7 females) in 53 traceable members have been living in Inner Mongolia of north China for 6 generations (Fig. 1). Audiology examination and physical examination were performed to confirm the clinical phenotypes. Medical history and life habit investigation of this family were also collected to rule out the possibility of environmentally associated deafness. Peripheral blood (10 ml) of each family member was drawn into two EDTA anticoagulant tubes and stored at the Institute of Genomic and Genetic Diseases for the Mongolian of Inner Mongolia University for the Nationalities for further study. The project was approved by the ethics committee of the Affiliated Hospital of Inner Mongolia University for the Nationalities. All participants signed informed consent.

### Clinical audiology test

Pure tone audiometry, tympanogram and acoustic reflection domain of the family members were examined in the Affiliated Hospital of Inner Mongolia Medical University to assess the hearing condition, middle ear conduction and nature of the disease.

### The criterion of hearing loss phenotype

As suggested by the proposal of EU hearing program [8], hearing loss was classified according to the average hearing threshold of the speech frequency: Normal (auditory threshold <20 dB HL), mild (auditory threshold of 20 - 40 dB HL), moderate (auditory threshold of 41 - 70 dB HL), severe (auditory threshold for 71 - 95 dB HL) and profound (auditory threshold>95 dB HL). According to the frequency distribution of hearing loss, it is divided into the hearing loss of high frequencies (2 - 8 kHz), low frequencies (less than or equal to 0.5 kHz), mid frequencies (0.5 - 2 kHz) and extended high frequencies (greater than 8 kHz).

### Gene detection

Whole genome sequencing: the whole genome sequencing and subsequent bioinformatic analysis of 5 family members, including 3 affected subjects and 2 normal controls, was carried out by Novogene Bioinformatics Institute, Beijing, China(Additional file 1: Tables S1–S2).

Sanger sequencing validation: the Sanger sequencing was used to verify the co-segregation of candidate pathogenic variant in members of the family with the deafness phenotype. The primers for PCR amplification of the pathogenic variant are: *OSBPL2* forward primer, 5 ‘-TCAGGTCCAGCGAAAATG-3, and *OSBPL2* reverse primer, 5’ -TTAGATGGGGAAAGGCAC-3′. The reaction conditions of PCR amplification are described as follows: 10 min pre-denaturation at 95 °C, 45 s denaturation at 94 °C, 45 s annealing at 50 °C, 45 s extension at 72 °C, for 35 cycles, and 72 °C extension 8 min after the end of the reaction. The PCR products were purified and sequenced by BGI-Beijing, Shenzhen, China.

### Blood lipid detection

The blood lipids including triglyceride and total cholesterol of the affected subjects and control individuals were tested at the Affiliated Hospital of Inner Mongolia Medical University. The Statistics of blood lipids was analyzed by single factor Logistic regression with Age normalization using SPSS.

## Results

### Phenotypic characteristics

**Fig. 2:**
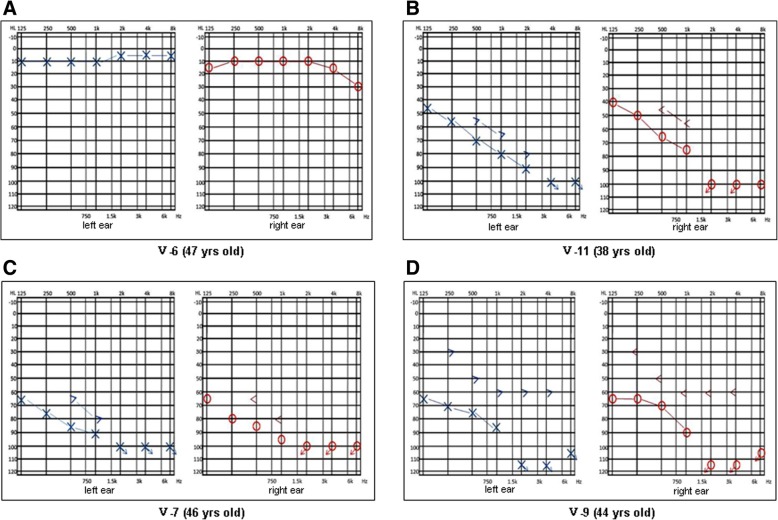
Pure tone audiometry of representative subjects. Both the left (blue symbol Χ, >, ↘) and right (red symbol Ο, <, ↙) ears of subjects were tested for their response to frequency (Hz, X-axis) and intensity (dB, Y-axis). Χ and Ο denote gas conduction; >and < denote bone conduction; ↘and ↙ denote no response; Audiograms of three different patients (V-7, V-9, V-11) are shown in **b**, **c**, **d** respectively and normal member (V-6) is shown in **a**. The sensorineural hearing loss ranges from mild (V-11), to moderate (V-9), and to severe (V-7)

The clinical manifestations of all deafness affected subjects were delayed hearing loss with the age of onset between 10 and 40 years old and a history of tinnitus but not vestibule dysfunction. All affected subjects had no history of ototoxic drug use, noise exposure, or mental disorders. All hearing loss was post lingual, and the severity increased with age. Hearing tests were diagnosed as bilaterally symmetric, mild to severe sensorineural hearing loss (Fig. 2). The results of tympanometry were normal (Additional file 2: Figure S1). Each generation of the family has affected members, both male and female, a characteristic of autosomal dominant deafness.

### Whole genome sequencing results

**Fig. 3:**
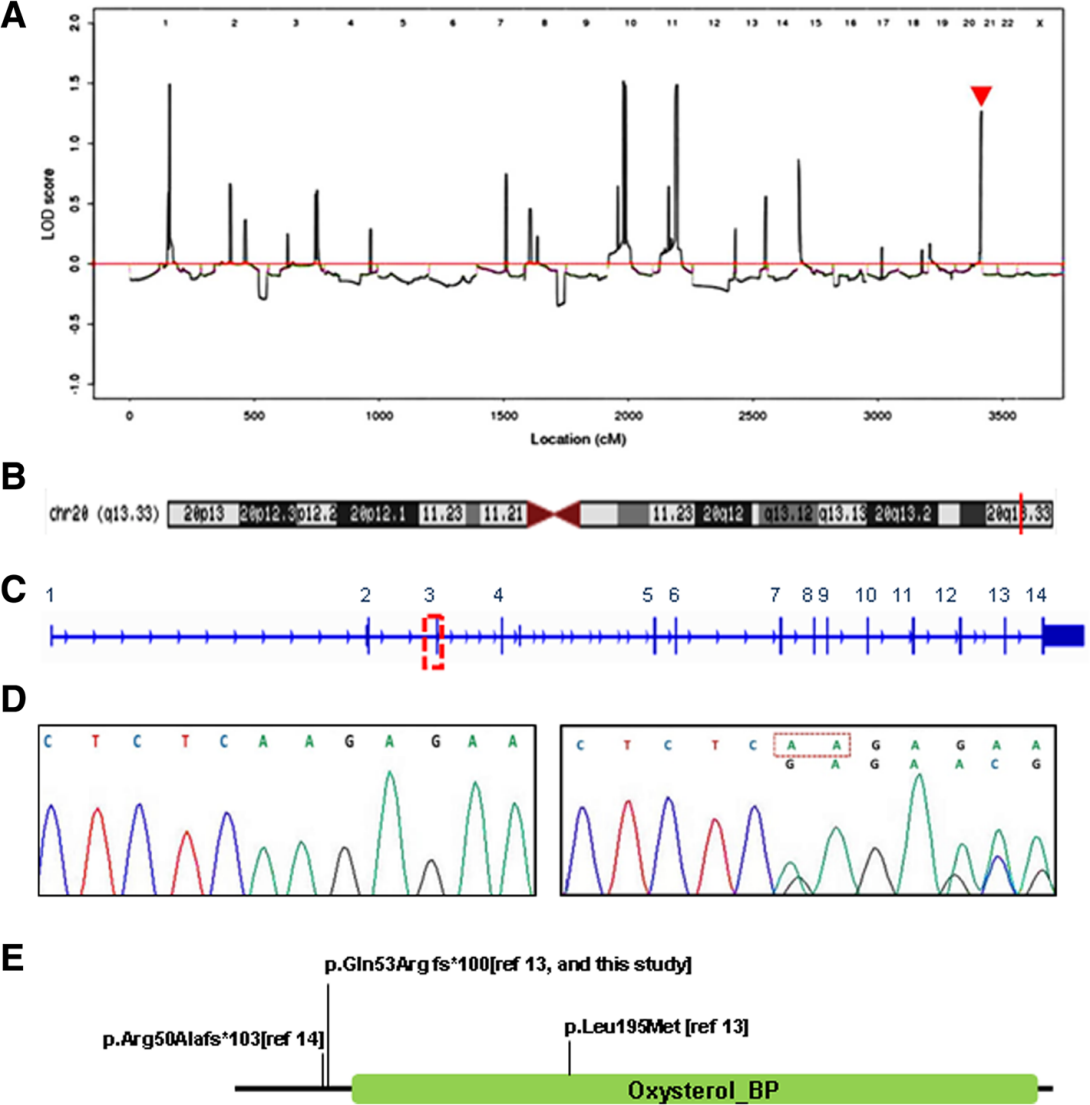
Genetic analysis of deafness family. **a** Linkage analysis detects four loci with LOD score around 1.5, red arrow points to the locus of *OSBPL2*. **b** *OSBPL2* is located on chromosome 20q13.33 (red bar). **c** The *OSBPL2* deletion is detected in exon 3 (red box). **d** Sequencing chromatograms of *OSBPL2* shows a heterozygous frameshift deletion c.158_159delAA (deleted nucleotides are boxed) in DNA from affected patients (right) as compared to that from control (left). **e** The c.158_159delAA (p.Gln53Arg fs*100) mutation of *OSBPL2* occurs before the oxysterol binding domain. Other published mutations on *OSBPL2* were also shown

Our initial attempt with exome capture sequencing on 127 known deafness genes failed to identify any pathogenic mutations (Additional file 1: Table S3).To identify the causal gene responsive for the hearing loss in this family, we conducted whole genome sequencing of 5 family members, including 3 affected subjects (IV-2, IV-9, and V-9) and 2 normal controls (IV-14 and V-6). Linkage analysis using Perl based Merlin tools was performed combining high throughput sequencing data in the family and the allelic frequency of Chinese population (CHB) in the HapMap database, and known SNP marker. Several linkage candidate regions were detected even though with low LOD score around 1.5 (Fig. 3).

All the SNPs and indels (insertion and deletion) detected by whole genome sequencing were filtered through 1000 Genome Project (ftp://ftp.1000genomes.ebi.ac.uk/vol1/ftp). The candidate genes were further screened by the in house precision algorithm of Novogene (Beijing, China) combined with the sequencing results and a variety of databases including Single Nucleotide Polymorphism database (dbSNP), Exome Aggregation Consortium (ExAC), Functional annotation of genetic variants from high-throughput sequencing data (esp6500siv2_all), ALL variants dataset of 1000 Genomes Project released in Aug 2015 (1000g2015aug_all). We then narrowed down the list of candidate genes to *OSBPL*2, considering the disease genotype and phenotype association. A frameshift mutation in *OSBPL*2, c.158_159delAA, was detected after whole genome sequencing which seems to be the pathogenic variant responsive for the hearing loss in this Mongolian family. *OSBPL*2 localizes 20q13.33 (www.genome.ucsc.edu), consistent with the peak detected on the chromosome 20 in linkage analysis (Fig. 3).

### Frameshift mutation validation by sanger sequencing

We next sought to confirm the frameshift mutation (c.158_159delAA) on *OSBPL2* associated with the hearing loss in this family. The target fragment spanning the frameshift mutation (c.158_159delAA) was amplified by PCR and purified with agarose gel electrophoresis (426 bp). The PCR products were then sequenced by Sanger sequencing. We chose to amplify 13 samples for Sanger sequencing validation. The frameshift mutation in *OSBPL*2, c.158_159delAA, co-segregated in the family and was only detected in patients with hearing loss (Fig. 1). In addition, this frameshift mutation in *OSBPL*2, c.158_159delAA, was negative from another 201 normal individuals who had been sequenced in our previous work (Chinese National Genebank CNSA, https://db.cngb.org/cnsa), further confirming that this frameshift mutation is the pathogenic variant leading to hearing loss in this Mongolian family.

### Blood lipid detection

*OSBPL2* encodes a receptor and is mainly involved in regulation of cellular cholesterol transport and lipid metabolism. To find out whether the frameshift mutation in *OSBPL2* gene affects lipid metabolism, we next tested the blood lipid level in members of this family with the confirmed genotype for *OSBPL2*. The results showed that the serum lipid levels of individuals with hearing loss are all within normal range, and are indistinguishable from those of normal individuals as determined by the *p* value (Table 1), suggesting the redundancy of other OSBP proteins in compensating the defect of *OSBPL2* in controlling blood lipid metabolism. However, this hypothesis needs to be further tested by knocking out of individual *OSBP* genes in mouse model or cell lines.

**Table 1:** The blood lipid level in members of this family

No.	Individual	Age (ys.)	Age of onset(ys.)	HDL-C (mmol/L)	TCh (mmol/L)	TG (mmol/L)	LDL-C (mmol/L)
1	IV-2	61–70	20	1.25	4.28	1.08	3.25
2	IV-9	61–70	14	1.30	5.50	1.07	3.80
3	IV-12	61–70	27	1.40	3.80	1.55	2.86
4	V-7	41–50	12	1.00	3.60	0.71	2.13
5	V-9	41–50	17	1.15	4.83	0.66	2.35
6	V-11	31–40	22	1.00	4.60	1.74	3.54
7	VI-5	21–30	19	1.14	3.22	0.59	1.81
8	IV-14	51–60	non affected	1.29	4.18	1.53	2.69
9	VI-6	21–30	non affected	1.70	3.90	0.90	2.00
10	V-6	41–50	non affected	1.10	5.90	1.68	3.80
11	V-13	31–40	non affected	1.50	5.24	1.02	3.25
12	VI-1	21–30	non affected	1.40	4.70	1.56	3.10
13	VI-4	11–20	non affected	1.65	5.50	1.47	3.50

Reference value: HDL-C: 0.9~1.7 mmol/L; TCh: 2.9~6.0 mmol/L; TG: 0.9~1.7 mmol/L; LDL-C: 2~4.11 mmol/L

The *p* value between affected and control group: HDL-C: 0.184; TCh: 0.128; TG: 0.259; LDL-C: 0.137. *p* < 0.05 is considered to be significant

## Discussion

Hereditary deafness is a common form of severe hearing impairment. In this study, a new frameshift mutation, c.158_159delAA (p.Gln53Arg fs*100) in *OSBPL2* (NM_144498.2), was found in a family with hereditary late-onset non-syndromic deafness by whole genome sequencing, followed by confirmation with Sanger sequencing. This frameshift mutation (c.158_159delAA) of *OSBPL2* results in abnormal OSBPL2 protein product with amino acid change starting from the 53rd amino acid and right before the sterol binding pocket on OSBP-related domain (aa75–470, http://pfam.xfam.org/protein/Q9H1P3), completely abolishing the binding capacity of mutant protein to sterol [9].

*OSBPL2* encodes a receptor with high affinity to oxidized sterols belonging to a family of oxysterol binding proteins (ORPs), which play an important function in sterol/lipid transport and metabolism, cell activity regulation and signal transduction. The pathogenic variants in the OSBP/ORP family members have been shown to cause diseases (such as dyslipidemia, cardiovascular disease) [10–12]. Pathogenic variant in the *OSBPL*2 gene has also been shown to cause autosomal dominant hereditary hearing loss. Genetic analysis of a Chinese autosomal dominant deafness family identified a frameshift mutation in *OSBPL*2 gene (c.153_154delCT) providing the first evidence that *OSBPL*2 is associated with the occurrence of non-syndromic deafness [13]. Subsequently, another frameshift mutation (c.141_142delTG) in the *OSBPL2* gene was also found in a German NSHL family, further confirming that *OSBPL*2 is a new deafness gene [14]. Interestingly, protein sequence change p.Gln53Arg fs*100 on OSBPL2 resulting from the frameshift mutation c.158_159delAA in this study is coincidentally identical to the reported c.153_154delCT [13].

Cholesterol is an important molecular component of mammalian plasma membrane and an important regulator for the properties of membrane. Recently, changes in cochlear cholesterol levels were shown to modulate the amplitude of distortion product otoacoustic emissions (DPOAEs), consistent with changes in electromotility [15]. Some studies have tried to explain the role of membrane cholesterol in the physiology of hair cells [15]. A recent study tried to connect the relationship between hearing loss and high cholesterol, and found that hearing level seems to be affected by hyperlipidemia [16].

OSBPL2 is mainly involved in the physiological functions of cell cholesterol transport and lipid metabolism. Over expression of *OSBPL2* in mammalian cells has been reported to alter cholesterol synthesis, cholesterol esterification and cholesterol efflux [17]. In this study, blood lipid examination found that serum lipid levels from both non affected and affected individuals are within the normal range and are not different by statistics. This could be due to the redundant expression of other *OSBP* genes and deficiency of a copy of *OSBPL2* gene does not shift the homeostasis of cholesterol metabolism in the digestive system.

*OSBPL2* is highly expressed in the inner and outer hair cells of the mouse cochlea [13, 14] and cholesterol level in hair cells seems to affect hearing [16]. It is plausible that *OSBPL2* is the major OSBP gene expressed in hair cells, which could be very sensitive to the dosage of *OSBPL2*. A frameshift mutation in the gene encoding OSBPL2 might completely block the transport and metabolism of cholesterol in the cochlear hair cells and therefore results in hearing loss. However, this hypothesis needs to be tested by examining the expression pattern of other OSBP/ORP family members in the cochlea as well as by studying animal models with *OSBPL2* deficiency.

## Conclusion

A frameshift deletion in *OSBPL2* (c.158_159delAA) was identified by whole genome sequencing as the pathogenic variant of a Mongolian family hereditary late-onset deafness. Our finding extends the pathogenic variant spectrum of *OSBPL*2 in autosomal dominant hereditary deafness (ADNSHL) and provides the genetic basis to include *OSBPL*2 in genetic counseling for deafness screening.

## Additional files

Additional file 1: **Table S1.** The genome-wide analysis list. **Table S2.** Average coverage. **Table S3.** 127 genes panel of hereditary deafness(in BGI). (PDF 80 kb)

Additional file 2: **Figure S1.** Results of tympanometry. (PDF 31 kb)

## Abbreviations

ADNSHL: Autosomal dominant hereditary deafness
DFNA: Autosomal dominantnon-syndromic sensorineural hearing loss
DPOAEs: Distortion product otoacoustic emissions
NSHL: Non-syndromic hearing loss
ORPs: Oxysterol binding proteins
OSBPL2: Oxysterol-binding protein-related protein 2
SHL: Syndromic hearing loss
