# Supplementary material for: A novel pathogenic variant in OSBPL2 linked to hereditary late-onset deafness in a Mongolian family
Source: BMC Med Genet. 2019 Mar 20;20:43. doi: 10.1186/s12881-019-0781-3 (PMC6425609; doi:10.1186/s12881-019-0781-3)
Supplement: Supplementary file 1 — Table S1. The genome-wide analysis list. Table S2. Average coverage. Table S3. 127 genes panel of hereditary deafness(in BGI). (PDF 80 kb) [file 12881_2019_781_MOESM1_ESM.pdf]

Additional file1

Table S1 The genome-wide analysis list

Analysis	software	Notes	version
comparison	BWA	The sequencing results were compared with the reference genome and the BAM results were obtained	0.7.8-r455
	SAMtools	Sort the results of comparison	
	picard	Combine the BAM results of the same sample and label repetitive read	1.111
SNP/INDEL testing	SAMtools	Detecting, filtering SNP and InDel information and separating the results	1.0
	ANNOVAR	Annotation SNP and Indel	2015Dec14
CNV testing	Conrol-FREEC	Test sample CNV	V7.0
	ANNOVAR	Annotation CNV	2015Dec14
SV testing	CREST	Test sample SV	V0.0.1
	ANNOVAR	Annotation SV	2015Dec14
Linkage area detection	Merlin	Detection of Linkage Regions in Family Samples	1.1.2

* ①Reagents for library preparation：NEBNext Ultra II DNA Library Prep

②Reagents for sequencing：HiSeq X HD Reagent Kit v2.5 (300 Cycles);

③Platform for sequencing：Illumina HiSeq X

Table S2 Average coverage

Report ID	1	2	3	4	5
**Sample:**[1]	**IV-9**	**V-9**	**IV-14**	**V-6**	**IV-2**
Total:[2]	610234924 (100%)	629292088 (100%)	622617482 (100%)	610312588 (100%)	601555452 (100%)
Duplicate:[3]	78151627 (12.85%)	76260918 (12.17%)	76907782 (12.39%)	78883413 (12.96%)	72839593 (12.15%)
Mapped:[4]	608220483 (99.67%)	626811417 (99.61%)	620732003 (99.70%)	608555836 (99.71%)	599626727 (99.68%)
Properly mapped:[5]	601939952 (98.64%)	620072528 (98.53%)	614816422 (98.75%)	601942230 (98.63%)	593644962 (98.68%)
PE mapped:[6]	607574190 (99.56%)	626038982 (99.48%)	620157972 (99.60%)	607853456 (99.60%)	598811774 (99.54%)
SE mapped:[7]	1292586 (0.21%)	1544870 (0.25%)	1148062 (0.18%)	1404760 (0.23%)	1629906 (0.27%)
With mate mapped to a different chr:[8]	2767140 (0.45%)	2790340 (0.44%)	2411268 (0.39%)	2901500 (0.48%)	2335354 (0.39%)
With mate mapped to a different chr ((mapQ>=5)):[9]	1876634 (0.31%)	1837614 (0.29%)	1589335 (0.26%)	2011286 (0.33%)	1518043 (0.25%)
Average_sequencing_depth:[10]	30.74	31.60	31.43	30.74	30.17
Coverage:[11]	99.81%	99.82%	99.07%	99.09%	99.80%
Coverage_at_least_4X:[12]	99.63%	99.64%	98.91%	98.91%	99.64%
Coverage_at_least_10X:[13]	98.41%	98.57%	98.49%	98.46%	98.39%
Coverage_at_least_20X:[14]	88.03%	89.48%	91.58%	91.00%	87.21%

Table S3 127 genes panel of hereditary deafness(in BGI)

Name	Detection of genes
Autosomal recessive nonsyndromic hearing impairment	*GJB2,GJB6,MYO7A,MYO15A,FOXI1,KCNJ10,SLC26A4,TMIE,TMC1,TMPRSS3,OTOF,CDH23,ATP2B2,GIPC3,STRC,OTOG,USH1C,TECTA,OTOA,PCDH15,RDX,GRXCR1,TRIOBP,CLDN14,MYO3A,DFNB31,ESRRB,ESPN,MYO6,GJA1,HGF,ILDR1,MARVELD2,DFNB59,SLC26A5,LRTOMT,LHFPL5,BSND,MSRB3,LOXHD1,TPRN,GPSM2,PTPRQ,SERPINB6,GJB3*
Autosomal dominant nonsyndromic hearing impairment	*ACTG1,CCDC50,CEACAM16,COCH,CRYM,DFNA5,DIABLO,DIAPH1,DSPP,EYA4,GJB2,GJB3,GJB6,GRHL2,KCNQ4,MIR96,MYH14,MYH9,MYO1A,MYO6,MYO7A,POU4F3,SIX1,SLC17A8,TECTA,TJP2,TMC1,WFS1,DIAPH3*
X-linked hereditary hearing impairment	*PRPS1,POU3F4,SMPX*
Maternally inherited hearing impairment	*MT-RNR1,MT-TS1*
Syndromic hearing impairment	*SERAC1,PDSS1,FGFR3,FGFR1,FGFR2,PHEX,DLX5,TNFRSF11B,COL2A1,COL11A1,COL9A1,COL9A2,COL4A3,COL4A4,COL4A5,BSND,SOX9,PAX2,GATA3,SLC19A2,IGF1,PAX3,MITF,SNAI2,EDNRB,EDN3,SOX10,HOXA1,SOBP,EYA1,SIX5,SIX1,CHD7,SEMA3E,SMAD4,FGF3,TCOF1,PRRX1,GLI3,HOXA2,KCNQ1,KCNE1,CACNA1D,ALMS1,LRP2,TIMM8A,NDP,WFS1,OPA1,SLC4A11,MYO7A,USH1C,CDH23,PCDH15,USH1G,USH2A,GPR98,PDZD7,DFNB31,CLRN1,MT-TK,MT-TE,MT-TL1,SLC26A4,KCNJ10,FOXI1*
