# Supplementary figures and images for: A novel pathogenic variant in OSBPL2 linked to hereditary late-onset deafness in a Mongolian family
Source: BMC Med Genet. 2019 Mar 20;20:43. doi: 10.1186/s12881-019-0781-3 (PMC6425609; doi:10.1186/s12881-019-0781-3)

## Additional file2

**Figure S1 Results of tympanometry**

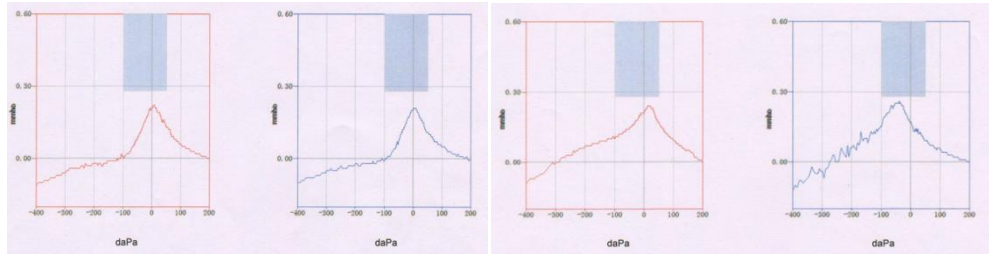

Supplement: Supplementary file 2 — Figure S1. Results of tympanometry. (PDF 31 kb) [file 12881_2019_781_MOESM2_ESM.pdf]
